# Translation and Cross-Cultural Adaptation of the Neck Dissection Assessment Tool to Spanish Language

**DOI:** 10.1055/s-0045-1810080

**Published:** 2025-10-09

**Authors:** Carlos M. Chiesa-Estomba, Miguel Mayo-Yanez, Jerome R. Lechien, Jon Alexander Sistiaga-Suarez, José Ángel González-García, Ehkiñe Larruscain, Laura Rodrigáñez-Riesco, Tareck Ayad

**Affiliations:** 1 Department of Head and Neck Surgery, Hospital Universitario Donostia, Biodonostia Research Institute, San Sebastián, Spain; 2 Head & Neck Study Group of Young-Otolaryngologists, International Federations of Oto-rhino-laryngological Societies (YO IFOS), Paris, France; 3 Department of Head and Neck Surgery, Complexo Hospitalario Universitario A Coruña (CHUAC), A Coruña, Spain; 4 Department of Otorhinolaryngology, Elsan Hospital, Poitiers, France; 5 Department of Otolaryngology – Head and Neck Surgery, La Paz University Hospital, Madrid, Spain; 6 Department of Otolaryngology – Head and Neck Surgery, Centre Hospitalier de l'Université de Montréal, Montreal, Québec, Canada

**Keywords:** assessment, competency, neck dissection, resident, translation, head neck, otolaryngology

## Abstract

**Introduction:**

Training in Head and Neck surgery represents a complex process always in evolution. Due to the absence of specific surgical evaluation tools to assess neck dissection performance by residents during training, finding a proper way to evaluate those standards will be a matter of debate for decades, and continues to be a pending task in the field.

**Objective:**

Validation and cross-cultural adaptation of the TSCND questionnaire to the Spanish language.

**Methods:**

A prospective data collection, about the performance of Spanish TSCND measured with Cronbach α.

**Results:**

Internal consistency of the task-specific checklist section measured was 0.752 (95% CI = 0.638 to 0.839) with an intraclass correlation coefficient of 0.749 (95% CI = 0.651–0.837). Internal consistency of the global rating scale section measured with Cronbach α was 0.788 (95% CI = 0.681 to 0.865) with an intraclass correlation coefficient of 0.789 (95% CI = 0.703–0.857).

**Conclusion:**

The development of specific tools to support surgical training education makes it possible to improve surgical skills evaluation. The Spanish translation of the TSCND is a reliable option for Spanish-Speaking surgical training and trainers in the development of an up-to-date competency-based curriculum.

## Introduction

Training in Head & Neck (H&N) surgery represents a complex process always in evolution. Finding a proper way to evaluate those standards will be a matter of debate for decades and continues to be a pending task in the field.

In some countries like the USA, an outcome initiative directed to collect reliable and accurate data that depicts the residents' ability to care for patients in the health care system was proposed in 1998, under the umbrella of the Accreditation Council for Graduate Medical Education (ACGME), requiring for US medical education programs to enhance assessment of resident performance during their training. 1

In Spanish-speaking countries, despite the search for the same criteria of excellence during residency training in Otolaryngology-Head and Neck Surgery (OTL – HNS), we still have a lack of reliable instruments to objectively evaluate resident competency acquisition. And like in other systems, verbal comments among supervising physicians on a case-by-case basis is the main form of feedback that residents will obtain. 2

Since its description by Crile in 1906, neck dissection (ND) remains one of the most relevant procedures during training in OTL-HNS residency programs. 3 In a recent systematic review conducted by Mercier *et al.* 4 the authors highlight the absence of specific surgical evaluation tools to assess neck dissection performance by residents during training.

Following these findings and considering the challenges and requirements of modern surgical education, Yang *et al* . developed the first tool assessing surgical competency in neck dissection. They provided preliminary evidence of feasibility, reliability, and validity of the Task Specific Checklist for Neck Dissection (TSCND). 5

The aim of this study is to document the reliability, validity, and cross-cultural adaptation of the TSCND in the Spanish Language.

## Methods

This study was approved by the Research and Ethics Committee (register code 2019/CCH011). The study was performed in accordance with the ethical standards laid down in the Declaration of Helsinki.

The TSCND is a questionnaire designed to evaluate resident's competency in neck dissection 5 inspired by the Objective Structured Assessment of Technical Skills (OSATS), commonly used in general surgery training. 6 This assessment tool uses a double checklist methodology: A global rating scale (GRS) which examines the resident's overall technical skills as well as fundamental surgical principles, and a task-specific checklist (TSC) which examines the resident's ability to perform each step of the procedure.

**Fig. 1:**
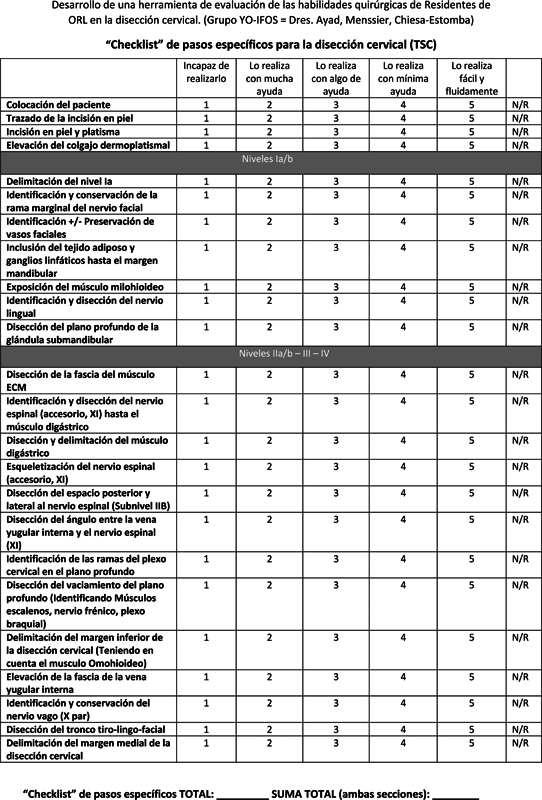
Global Rating Scale (GRS).

**Fig. 2:**
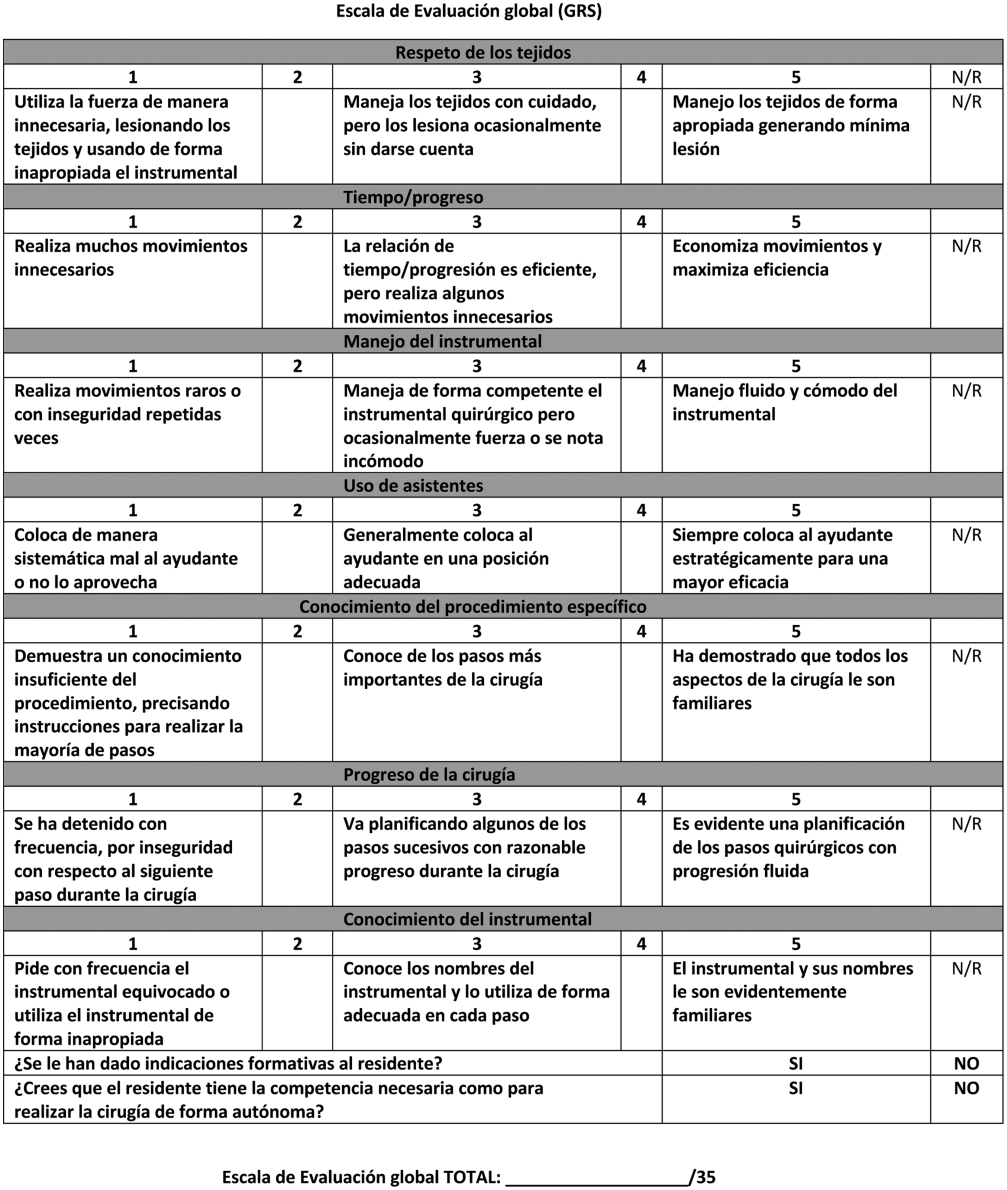
Task Specific Checklist (TSC).

We followed the recommendations of the World Health Organization for the translation and adaptation of instruments and the cross-cultural adaptation of health-related quality of life measures proposed by Guillemin *et al* , 7 the following way: Stage I: first translation was carried out by two independent Otolaryngologists - Head & Neck Surgeons, Spanish English native speakers. No consensus was needed since the two translations were perfectly similar. Stage II: After translation the survey was sent to a panel of six experts in Head and Neck Surgery throughout Spain and Latin-America. Stage III: After consensus was reached, the definitive version was sent to a third bilingual English Spanish speaker Otorhinolaryngology - Head & Neck Surgeon for back-translation. Afterward, Stage IV: the comprehensibility of the translated questionnaire was evaluated in a group of residents to check the reliability of the questionnaire. Stage V: The definitive version of the questionnaire obtained was finally reviewed again by a panel of experts and a consensus was obtained ( Figs. 1 & 2 ).

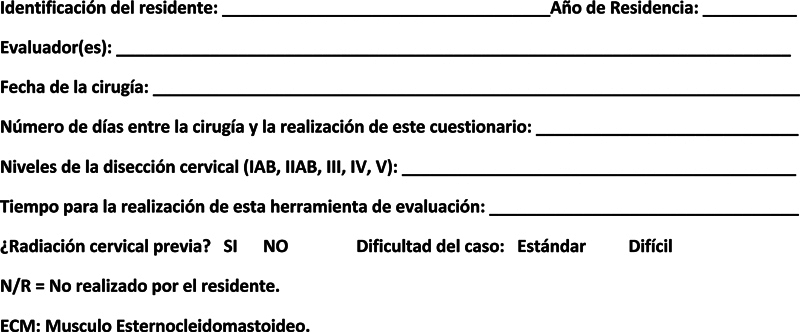



To assess the reliability of the Spanish translation of this questionnaire, five 3 ^rd^ year residents (PGY-3) and five 4 ^th^ year residents (PGY-4) from 2 different hospitals were evaluated during their H&N training rotation, prospectively by 6 different consultants between January 15th of 2020 and January 15th of 2022, when at least 2 different consultants evaluate the same resident on each single surgery. First and second-year residents were excluded given their limited involvement in neck dissection in the departments included. All residents and consultants agreed to participate in the validation process. During all the process, the evaluations remain confidential and written consent was obtained from each participant.

As in the validation study, assessing the feasibility of implementing the TSCND time needed to complete the evaluation was recorded in minutes. Furthermore, the time delay between the day of the surgery and the day the scoring grid was completed was recorded in days.

Neck dissection was performed according to common practice. Dissection of levels IIa and IIb, III and IV were the most often performed during the study period.

Statistical analysis was conducted with SPSS for Macintosh Version 21.0 (IBM Corp, Armonk, NY). Internal consistency was measured with Cronbach α and temporal stability by using the intraclass correlation coefficient (P < 0.05). To analyze potential differences between groups with continuous variables, we performed the Wilcoxon test for nonparametric data and student's t-test for parametric data. Differences between groups with categorical variables were computed by Chi-square test and Fisher's exact test. A p value <0.05 was considered significant.

In the psychometric evaluation of the instruments, Reliability refers to consistency or dependability after repeated measurements. Consistency, to evaluate which each question of the scale relates to the rest, using the Cronbach α method to measure it, and taking a value over 0.70 to suggest a strong construct validity, which gets better the closer it is to 1. And finally, Temporal Stability, known as test-retest reliability, was performed after 8 weeks; and it refers to the concordance between the scores of repeated measurements from the same participant. It can be assessed by the intraclass correlation coefficient, considering 0.70 as a good correlation value.

An exploratory factor analysis (EFA) was conducted to assess the underlying structure of the Spanish version of the TSC and GRS for Neck Dissection. Due to the exploratory nature of the study and the lack of prior evidence regarding the dimensionality of the instrument in the Spanish-speaking context, EFA was deemed appropriate. Prior to the analysis, raw item scores were screened, and non-numeric responses (e.g., "n/c") were treated as missing values. The data were standardized to ensure comparability across items. The factor analysis was performed using Principal Axis Factoring, and the number of factors to retain was determined based on the Kaiser criterion (eigenvalue > 1) and visual inspection of the scree plot. All analyses were conducted using Python 3.9 with the sklearn library.

## Results

A total of 40 neck dissections (ND) were performed by 10 residents and evaluated by six supervising surgeons using the TSCND during the study period. Of these, 20 dissections (50%) were performed by five PGY-3 residents, and the remaining 20 (50%) by five PGY-4 residents. On average, each resident performed four neck dissections during the study period. Key steps such as identifying the accessory nerve (cranial nerve XI) and the posterior belly of the digastric muscle, dissecting level IIb, and addressing vascular structures were most strongly associated with higher overall scores.

Regarding the overall performance, the TSCND-TSC score represents the technical skills assessment for ND. The mean score across all residents was 60.2 points. Breaking this down by year of training, PGY-3 residents scored an average of 55.4 points, while PGY-4 residents achieved a mean score of 65 points. This difference was statistically significant (p = 0.001), indicating that more advanced training was associated with better technical performance.

The TSCND-GRS score reflects a general rating scale used to evaluate overall performance during the procedure. The mean score for all residents was 27.6. For PGY-3 residents, the average was 27.7 points, compared to 30.6 points for PGY-4 residents. However, this difference was not statistically significant (p = 0.228), suggesting that the variation in general performance between the two groups was less pronounced.

The Cronbach's α was used to assess internal consistency and reliability. When we used this test, values closer to 1 indicate that the test items measure the same underlying construct. For the TSC section, Cronbach's α was 0.752 (95% CI: 0.638–0.839), which suggests a good level of reliability. After removing individual test items, the α value remained above 0.731, indicating that no single item disproportionately influenced the test's reliability. Items related to defining the inferior and medial boundaries of the neck dissection showed particularly strong contributions to reliability ( Table 1 ).

**Table 1:** Consistency and Interclass Correlation Coefficient of the Spanish Version of the TSCND, subsection Task Specific Checklist (TSC) measured with Cronbach α

	Total	PGY-3	PGY-4
Cronbach α	0.752	0.786	0.744
Positioning of patient	0.749	0.780	0.701
Positioning of skin incision	0.755	0.790	0.754
Incision of skin and platysma	0.753	0.771	0.691
Elevation of subplatysmal flap	0.752	0.767	0.694
Delimitation of level IA	0.703	0.739	0.713
Identification and preservation of marginal mandibular nerve	0.708	0.734	0.724
Identification +/- preservation of facial vessels	0.741	0.700	0.699
Inclusion of adipose tissue and lymph nodes up to the mandibular margin	0.713	0.709	0.732
Expose and elevate mylohyoid muscle	0.703	0.736	0.713
Identification and freeing of lingual nerve	0.702	0.733	0.711
Dissection of the deep plane of the submandibular gland	0.699	0.731	0.731
Unwrapping fascia from SCM muscle	0.752	0.787	0.716
Identification and dissection of accessory nerve (XI) up to digastric muscle	0.749	0.784	0.733
Dissection and delimitation of digastric muscle	0.754	0.797	0.721
Skeletonize accessory nerve	0.752	0.792	0.719
Dissection of retrospinal level (IIB)	0.749	0.784	0.759
Dissection of the angle between the internal jugular vein and the accessory nerve (XI)	0.748	0.794	0.728
Identification of branches of the deep cervical plexus	0.752	0.792	0.702
Resection of the specimen from the deep plane	0.755	0.788	0.748
Delimitation of the inferior margin of the dissection	0.769	0.806	0.781
Unwrapping fascia from the internal jugular vein	0.754	0.790	0.765
Identification and preservation of the vagus nerve (X)	0.744	0.767	0.715
Dissection of the thyroid-lingual-facial trunk	0.751	0.787	0.716
Delimitation of the medial margin of the dissection	0.759	0.796	0.756
Interclass correlation coefficient	0.749 (95% CI = 0.651–0.837).	0.787 (95% CI = 0.650 to 0.889)	0.709 (95% CI = 0.549 to 0.749)

The intraclass correlation coefficient (ICC) measures agreement or consistency between evaluators. For the TSC section, the ICC was 0.749 (95% CI: 0.651–0.837), indicating substantial agreement among evaluators.

When we perform the subgroup analysis by residency year, the internal consistency of the TSC section was analyzed separately for each training year. For PGY-3 residents, Cronbach's α was 0.786 (95% CI: 0.658–0.879), and the ICC was 0.787 (95% CI: 0.650–0.889). For PGY-4 residents, Cronbach's α was 0.744 (95% CI: 0.540–0.782), and the ICC was 0.709 (95% CI: 0.549–0.749). These values indicate good reliability for both training groups. Removing individual test items did not significantly reduce reliability, with minimum Cronbach α values of 0.700 for PGY-3 residents and 0.691 for PGY-4 residents ( Table 1 ).

The GRS section also showed strong internal consistency, with a Cronbach's α of 0.788 (95% CI: 0.681–0.865). This suggests that the items in the GRS reliably evaluate general surgical performance. Even after removing items, the α value remained above 0.759, with the highest reliability for items assessing assistant support and surgical progress ( Table 2 ). The ICC for the GRS section was 0.789 (95% CI: 0.703–0.857), reflecting high agreement among evaluators.

**Table 2:** Consistency and Interclass Correlation Coefficient of the Spanish Version of the TSCND, subsection Global Rating Scale (GRS) measured with Cronbach α

	Total	PGY-3	PGY-4
Cronbach α	0.788	0.853	0.718
Respect of tissue	0.725	0.769	0.701
Time and motion	0.762	0.766	0.706
Instrument handling	0.742	0.800	0.699
Use of Assistants	0.796	0.831	0.724
Knowledge of specific procedure	0.758	0.801	0.711
Flow of operation	0.796	0.843	0.736
Knowledge of instruments	0.737	0.758	0.703
Interclass correlation coefficient	0.789 (95% CI = 0703–0.857)	0.823 (95% CI = 0.698 to 0.910)	0.713 (95% CI = 0.578 to 0.774)

For PGY-3 residents, Cronbach's α for the GRS section was 0.853 (95% CI: 0.751–0.954), indicating excellent reliability. The ICC was 0.823 (95% CI: 0.698–0.910), reflecting high consistency among evaluators. For PGY-4 residents, Cronbach's α was 0.718 (95% CI: 0.529–0.798), and the ICC was 0.713 (95% CI: 0.578–0.774), indicating good reliability. Also, removing items did not significantly affect consistency, with minimum Cronbach α values of 0.758 for PGY-3 residents and 0.699 for PGY-4 residents. These results demonstrate that the GRS is a reliable tool for evaluating performance across different levels of training ( Table 2 ).

Exploratory factor analysis revealed that the Spanish version of the TSCND exhibited a unidimensional structure. The scree plot and eigenvalue analysis (Kaiser criterion) indicated that only one factor had an eigenvalue greater than 1, supporting the retention of a single factor. This factor accounted for most of the variance among the items. All items demonstrated acceptable loadings on factor analysis, with most items exceeding 0.6, indicating strong contributions to the latent construction of technical performance in neck dissection. Items such as "Conservación de la rama marginal" and "Area I" presented particularly high loadings, highlighting their relevance within the checklist framework. ( Table 3 ) Exploratory factor analysis was performed also to examine the dimensionality of the GRS. The analysis revealed that the eigenvalue criterion and scree plot supported the retention of a single factor. However, the factor loading varied across the items. "Conocimientos del procedimiento especifico" showed the highest loading (0.79), indicating a strong contribution to the latent factor, presumably representing overall surgical competence. The remaining items, including "Respeto a los tejidos," "Tiempo/Progreso," "Manejo del instrumental," and "Uso de asistentes," exhibited lower loadings (range 0.22–0.34), suggesting a more moderate or weaker contribution to the underlying construct. These findings imply that while the GRS can be considered to measure an overarching domain of surgical competence, some items contribute less strongly, highlighting potential areas for refinement or further validation in larger samples. ( Table 4 )

**Table 3:** Factor analysis of the Spanish Version of the TSCND, subsection Task Specific Checklist (TSC)

Item	Factor Analysis	Interpretation
**Trazado incision**	−0,519	Weak or moderate loading
**Incision en piel**	−0,718	Strong loading (>0.6)
**Elevación colgajo**	−0,692	Strong loading (>0.6)
**AREA I**	−0,886	Strong loading (>0.6)
**Conservación marginal**	−0,924	Strong loading (>0.6)
**Exposición Milohioideo**	−0,94	Strong loading (>0.6)
**Identificación N. Lingual**	−0,95	Strong loading (>0.6)
**Disección Plano Profundo G. Submaxilar**	−0,922	Strong loading (>0.6)
**Disección Fascia ECM**	−0,8	Strong loading (>0.6)
**Identificación Espinal**	−0,78	Strong loading (>0.6)
**Musc. Digastrico**	−0,765	Strong loading (>0.6)
**Esqueletización Espinal**	−0,374	Weak or moderate loading
**Disección Iib**	−0,478	Weak or moderate loading
**Disección VYI y Espinal**	−0,373	Weak or moderate loading
**Identificación plexo cervical profundo**	−0,443	Weak or moderate loading
**Disección en el plan o profundo**	−0,592	Weak or moderate loading
**Delimitación margen inferior de la disección cervical**	−0,251	Weak or moderate loading
**Elevación fascia VYI**	−0,495	Weak or moderate loading
**Identifiación y conservación X par**	−0,532	Weak or moderate loading
**Disección TTLF**	−0,362	Weak or moderate loading
**Delimitación margen medial**	−0,734	Strong loading (>0.6)

**Table 4:** Factor analysis of the Spanish Version of the TSCND, subsection Global Rating Scale (GRS)

Item	Factor Analysis	Interpretation
Respeto a los tejidos	0,348	Weak or moderate loading
Tiempo/Progreso	0,33	Weak or moderate loading
Manejo del instrumental	0,223	Weak or moderate loading
Uso de asistentes	0,343	Weak or moderate loading
Conocimientos del procedimiento especifico	0,794	Strong loading (>0.6)
Progreso de la cirugía	0,463	Weak or moderate loading
Conocimiento del instrumental	−0,275	Weak or moderate loading

Finally, the average time required to complete the TSCND evaluation was 3.8 minutes (SD: 3.20), making it a quick and efficient tool. Supervising consultants typically completed their evaluations within two days of the surgical procedure (mean: 2 days, SD: 2.23).

## Discussion

Neck dissection is considered a diagnostic and therapeutic procedure in H&N cancers patients, that allows eradication of regional lymph node metastasis. Previous data suggests that ND offers an effective and oncological safe surgical procedure in selected patients with clinically positive metastatic nodes in the neck. 8 9 However, teaching and evaluation methods are different and mostly empirical across the world as well as work-hour restrictions, limitations about surgical availability, burnout or pressure related to clinical productivity make mandatory to design innovative assessment strategies.

Usually, surgical training deficiencies are identified at the end of training or in the first years of practice as a consultant when those are more difficult to improve. The TSCND was the first standardized method designed to assess resident competency in ND and track their progress during the training program. According to the best of our knowledge, this is the first study designed to perform a translation, validation, and cross-cultural adaptation of the TSCND from English to Spanish.

More recently, Dowling et al. 10 focused on selective neck dissection and designed for the evaluation of trainees according to the PGY-Level and the number of H&N surgical rotations. According to the authors, identification of key vascular structures as well as the omohyoid muscle and the posterior belly of the digastric muscle were the most strongly associated factors with the overall score, results in accordance with those observed in our cohort.

About the psychometric evaluation of the translated test, we demonstrated a good internal consistency of the TSCND-TSC and the TSCND-GRS over 0.7, a high specific year of training related consistency, also higher than 0.7 on both and a good intraclass correlation coefficient over 0.7 in all measures. Our results suggest that the questionnaire its reliable and useful for application in H&N Spanish-Speaking teaching environments. ( Table 1 & 2 ). Moreover, the results of our factor and Kaiser analyses reinforce the robustness and reliability of the TSC and GRS evaluation frameworks in assessing surgical performance. Both analyses revealed that a single dominant factor accounts for most of the variance in each dataset, emphasizing the coherence of the assessment items and their alignment with a unified construct of surgical competence. The strong contributions of Total and PGY-3 scores highlight the reliability of these measures, while the moderate influence of PGY-4 scores suggests developmental progression in skill acquisition. These findings validate the use of TSC and GRS as effective tools for evaluating residents' technical skills and overall performance across different training levels, providing a solid foundation for refining surgical education and assessment methodologies. ( Table 3 & 4 )

Results obtained in this analysis are like previously published data by Mercier et al. 11 and Yang et al., 5 in the two first publication about the use of the TSCND and allow us to suggest implementation of this tool. As we mentioned above, consistency of each item was good when comparing the evolution from PGY-3 to PGY-4 in our cohort. This also corroborates that the TSCND may be able to measure a difference in scores between groups that are expected to have unequal surgical abilities. Regarding the need to fill both checklists, it is important to understand that each checklist provides different feedback. The TSCND-GRS evaluates fundamental surgical abilities, whereas the TSCND-TSC evaluates the resident's proficiency in each step of ND.

Regarding surgical and educational purposes, the application of methods like this will be interesting for resident evaluation. However, some major obstacles need to be overcome. The need for surgical time, faculty compromise, and coordination with different trainees' schedules and department logistics requires a significant compromise. It is here, where integration of these methods into the surgical curriculum will facilitate the participation in activities such as this.

The translation of this test was done in Spain. Even though Latin American Spanish medical and surgical terms is almost the same as mainland Spain Spanish. However, the main author and some reviewers of this translated version of TSCND are from Latin America and the Caribbean, so words and expressions used in these countries were used.

Finally, there are some issues regarding the application of the TSCND that need to be considered: 1) participating assessors using the tool in this study were not blinded about the residents' identity and level of training; 2) the small sample size due to the limited number of residents available on each participating department 3) the variability in the complexity of neck dissections that may impact scoring, and 4) the lack of surgical skill and instrumentation evolution evaluation using this method during the training process. In this vein, further studies are required to validate whether this discriminates competence or improvement over time, that no correlation can currently be drawn to patient outcomes.

## Conclusion

In modern medicine, teaching methods are evolving. The development of specific tools to support surgical training education makes possible to improve case discussion between mentors and learners to stimulate individualized support. Due to the relevance of the Spanish Language, still the second most spoken language regarding the number of native speakers, the TSCND represents an option for Spanish-Speaking surgical training and trainers in the development of an up-to-date competency-based curriculum.
